# Comment on “Efficacy of Alendronate for Preventing Collapse of Femoral Head in Adult Patients with Nontraumatic Osteonecrosis”

**DOI:** 10.1155/2015/354109

**Published:** 2015-05-12

**Authors:** Hengfeng Yuan

**Affiliations:** Department of Cancer Biology, Mayo Clinic, No. 4500 San Pablo Road, Jacksonville, FL 32224, USA

I read with great interest the review by Hong et al. [1] and have the following comments to offer.

As a meta-analysis, the literature selection is a very important step. Failure to pick up the suitable one could lead to a bias or even a totally wrong conclusion in the article. In this review, I found that the authors wanted to include the randomized controlled trial (RCT); however, one of the included trails [2] is not an RCT but a prospective comparative study with Level II. Moreover, the purpose of this review was to study the efficacy of alendronate, but another included trial [3], in which the two compared groups were extracorporeal shock wave treatment (ESWT) versus ESWT + hyperbaric oxygen therapy (HOT) + alendronate treatment still needs to be further checked. It seems inappropriate to include this trial for the efficacy of alendronate could not be obtained alone but at least combined with HOT. Hence, I think both the authors and the reviews should be careful when they deal with such kind of research.
